# Recurrence of Cryptococcal Meningitis and the Hidden Role of Patient Education and Social Support

**DOI:** 10.1155/2018/8125096

**Published:** 2018-08-08

**Authors:** Felix Bongomin, Lorna Atikoro

**Affiliations:** ^1^Department of Medical Microbiology & Immunology, Faculty of Medicine, Gulu University, P.O. Box 166, Gulu, Uganda; ^2^Department of Medicine, Gulu Regional Referral Hospital, P.O. Box 180, Gulu, Uganda

## Abstract

Human immunodeficiency virus- (HIV-) associated cryptococcal meningitis (CM) is one of the leading causes of deaths among patients living with HIV/AIDS in resource-limited settings, accounting for ~15-20% of AIDS-related deaths globally. We present our experience with a 25-year-old woman living with HIV who had a recurrent cryptococcal disease due to nonadherence to HIV care and lack of awareness of the benefits of adherence to secondary prophylaxis for CM. This case highlights the fact that fungal diseases awareness should not be limited only to the health professionals and public health practitioners, but also to patients, caregivers, and stakeholders.

## 1. Introduction

Cryptococcal meningitis (CM) is a community-acquired opportunistic fungal infection of the central nervous system caused by* Cryptococcus* species of yeasts. CM is the second most common HIV-associated opportunistic infection worldwide and has become one of the leading causes of death among patients living with HIV/AIDS in resource-limited settings, causing ~15-20% of AIDS-related deaths globally [1]. About 73% of the over 200,000 estimated global annual cases of CM occur in Sub-Saharan Africa [2, 3].

Unlike bacterial and viral meningitis, adjunctive corticosteroid therapy is contraindicated for the management of CM and treatment is always long-term consisting of three phases: 2 weeks of induction therapy with amphotericin B plus flucytosine, and a further 8-10 weeks of consolidation with fluconazole 400-800mg daily followed by long-term fluconazole (200mg daily) therapy for maintenance phase, also known as secondary prophylaxis for at least 1 year [4–6]. The most recent evidence from the Advancing Cryptococcal meningitis Treatment for Africa (ACTA) randomised clinical trial suggests that short-course induction therapy (amphotericin B for 7 days + fluconazole or flucytosine) is safer and more efficacious than the current standard of care (amphotericin B for 14 days plus fluconazole or flucytosine) [7]. Short-course induction therapy is now the World Health Organization (WHO) preferred regimen [8]. Without secondary prophylaxis, recurrence of cryptococcal meningitis occurs in nearly half of the patients [9]. Previous studies have shown that relapses of symptomatic CM are often associated with fluconazole resistance and immune reconstitution inflammatory syndrome [10]. However, a recent systematic review suggest that fluconazole resistance does not explain all cases of CM relapses [11].

We present our experience with a patient who had a recurrent cryptococcal disease due to nonadherence to HIV care and lack of awareness of the benefit of adherence to secondary prophylaxis for CM.

## 2. Case

In February 2017, a Good Samaritan brought a 25-year-old waitress into the medical emergency unit of Gulu Regional Referral and Teaching Hospital (GRRTH) after she was found unconscious. By physical examination, she was well hydrated with full-volume peripheral pulses with no pallor of the conjunctivae or peripheral edema. Her Glasgow coma scale was 11/15, and signs of meningeal irritation were positive by Kernig's technique; however, pupils were equal, accommodative, and reactive to light. Her blood pressure was 115/70mmHg, pulse rate was 100 beats per minute, respiratory rate was 16 breaths per minute, and her body temperature was 37.5°C. Rapid diagnostic test for malaria was negative and random blood sugar was within normal limits. She was stabilized and managed conservatively. After a couple of hours at the emergency unit, she gained consciousness and was able to share her history of current illness. She reported progressive worsening of a headache, with no history of convulsions, fever, or visual disturbances. Review of other systems was noncontributory.

In her past medical history (PMH), she was diagnosed with HIV infection in November of 2016 following an admission at GRRTH for an acute illness. She had presented with CM as her index opportunistic infection. She reported a history of a severe headache and fever for about 3 weeks prior to her admission. Diagnosis of CM was achieved through examination of Cerebrospinal fluid (CSF) following a diagnostic lumbar puncture (LP). She was treated with fluconazole 1200mg daily for 2 weeks and then discharged home on 800mg daily by mouth. During this admission, she was also initiated on antiretroviral therapy (ART) consisting of Tenofovir/Lamivudine/Efavirenz. A follow-up date was given a month from the date of discharge. However, she did not return to the hospital on the given follow-up date, as she did not have the transport money to come to the hospital. Since then, she discontinued fluconazole therapy and was not also on ART.

**Figure 1:**
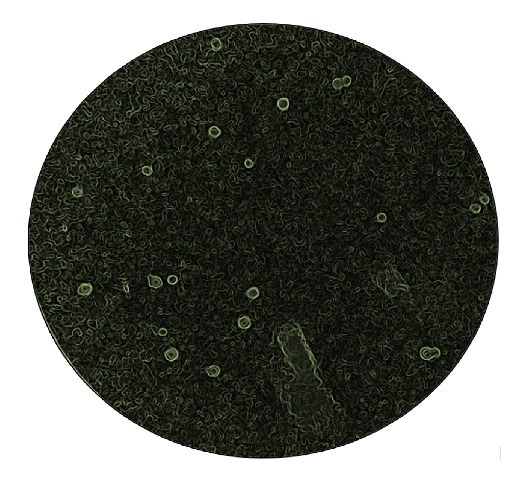
India ink preparation demonstrating numerous budding yeast cells surrounded by capsular halo consistent with the identification of* Cryptococcus* species. X100.

From her presentation and PMH, a recurrence of CM was the most likely diagnosis. Diagnostic LP was done to obtain CSF for analysis; serum and CSF point-of-care lateral flow assays were both positive for cryptococcal antigen (CrAg: Immuno-Mycologics [IMMY], Oklahoma, USA). Microscopic examination of an India ink preparation of the CSF demonstrated numerous budding yeast cells [Figure 1] consistent with morphologic identification of* Cryptococcus* species, confirming the clinical diagnosis. CSF fungal culture was not done.

Since amphotericin B was “out-of-stock” and flucytosine is not available in Uganda, for induction phase, she was treated with high dose (1200mg) fluconazole and she also received daily therapeutic LPs over 72 hours for the management of her assumed increased intracranial pressure (deferred from her very severe headaches). She reported some improvement after 2 weeks of treatment. Fluconazole dose was reduced to 800mg daily as per the national CM treatment guidelines. A repeat HIV antibody test was positive, she had a CD4 count of 79 cells/*μ*L, and her blood samples were sent for viral load assay at the Uganda Virus Research Institute, Entebbe. She was recommenced on ART in the 5^th^ week of her CM treatment.

**Figure 2:**
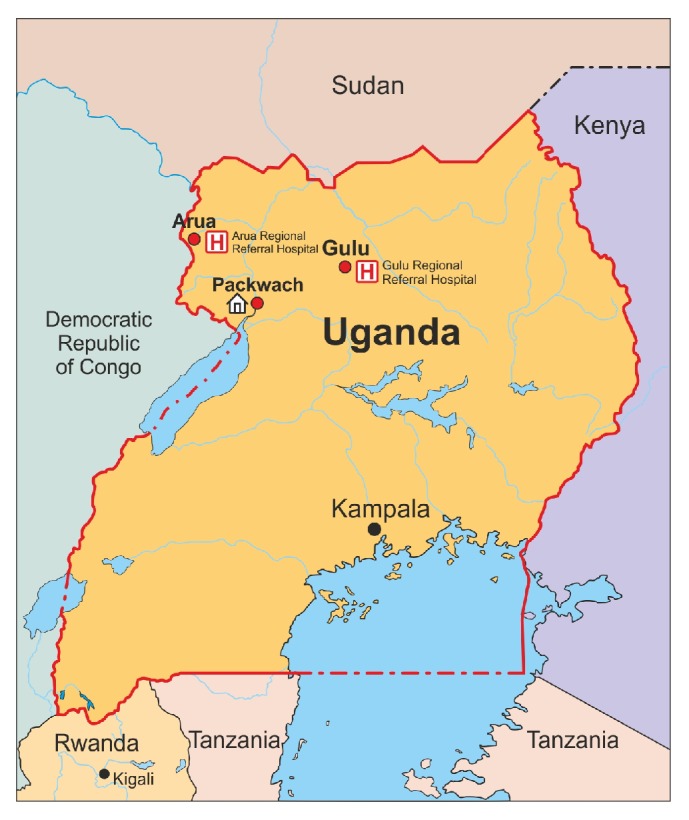
A map of Uganda showing the patient's hometown and the two regional referral hospitals where she could access HIV and cryptococcal meningitis care from.

She was not aware of the cause, treatment options, and duration and the need for long-term/life-long suppression therapy for her condition. She comes from Packwach, West Nile (~120KM from Gulu) [Figure 2], and was working as a bar waitress to earn a living in Gulu. She had no caretaker and did not have money to take care of her basic needs while in the hospital. She mostly relied on the support of good Samaritans and the support of other patient's caretakers for food and support. Through connections with another patient's caretaker, her brother was contacted and he requested that she is discharged and transferred for further care at Arua Regional Referral Hospital (~107KM from Pakwach) [Figure 2], as they did not have the financial resources to sustain her in Gulu. We lost contact with her thereafter.

## 3. Discussion

Long-term (at least 12 months) or life-long secondary prophylaxis with fluconazole or an alternative antifungal agent is always required, and it is associated with reduction in the rate of recurrence of CM from about 50% to under 5% [9]. The present case study adds to the evidence that reasons for recurrent disease is multifactorial and goes beyond clinical, microbiological, or pharmacological (treatment) realm. A well-tailored patient counseling and education and social support including home visits improves awareness, increases adherence, and reduces disengagement rate from chronic HIV/CM care. Adapted adherence support has recently been included in the WHO advanced HIV disease package of care [12].

Our patient had several reasons to explain the recurrence of her CM including, suboptimal therapy, poor adherence and compliance, lack of social support, and economic challenges. Importantly, disengagement from both CM and HIV care due to the above challenges contributed to disease progression in this case. Bicanic and colleagues reported that symptomatic relapse of HIV-associated CM is common after initial fluconazole monotherapy with an associated 6-month mortality in excess of 50% [10].

Emerging evidence from an ongoing trial suggests that weight based oral fluconazole dose of 1600mg is more effective than the current WHO recommended dose (1200mg) and safer than 2000mg but less effective than amphotericin B across all doses [13]. It is clear that amphotericin B based induction is the treatment of choice as per current treatment guidelines based on the best available evidence. Moreover, the ACTA trial showed that 2-week induction therapy with all-oral regimen (fluconazole and flucytosine) is as effective as short-course amphotericin B based regimen. Advocacy should be put across to make flucytosine available in areas of dire need as it has been shown to have super early fungicidal activity especially as a partner drug to amphotericin [7].

Despite ART expansion, in many parts of the world, the morbidity and mortality attributable to cryptococcal disease are largely unchanged in low-income and middle-income countries [14, 15].* Cryptococcus* is the most common cause of meningitis in Uganda both in patients newly diagnosed with HIV and in known HIV-infected patients who are not on ART [16]. Improved management (treatment and prophylaxis) of CM among HIV/AIDS patients receiving ART is essential to reducing ongoing AIDS mortality [16]. Effective induction therapy requires potent fungicidal drugs (amphotericin B and flucytosine) [7, 17], which are often unavailable in low-resource, high-endemicity settings. As a consequence, mortality is unacceptably high [18]. All antifungal agents for the treatment of CM are on the WHO Essential Medicine List. Fluconazole is available in almost all countries across the world. However, amphotericin B and flucytosine remain unavailable in many parts of Sub-Saharan Africa [19].

The most common causes of recurrent symptoms and signs of CM are nonadherence to consolidation fluconazole therapy or secondary prophylaxis, nonadherence to ART, mycological relapse (with a positive CSF culture of* Cryptococcus*), and paradoxical cryptococcal-immune reconstitution inflammatory syndrome [10, 20]. Compliance is dependent on an unlimited supply of antifungal agents for the entire period of secondary prophylaxis (12 months or more); these require a strong socioeconomic support to the patients and the ability of the patients to reach the health facilities. Public health and clinical attention are mostly on optimization of antifungal therapy and screening for asymptomatic antigenaemia [21]; very limited or no attention has been given to the social and economic challenges faced by these patients.

This case is of particular interest as it does not only support the growing evidence that a considerable proportion of patients represent to care with advanced HIV disease and life-threatening opportunistic infection [22], but also that multiple factors are associated with poor clinical outcomes. The roles of social support and patient education in recovery are the most neglected of these factors. We urge that fungal diseases awareness should not be limited only to the health professionals and public health practitioners, but also to patients and stakeholders. Educating the patient on the most relevant information including prognosis of CM and the need for long-term secondary prophylaxis, establishment of patient social support groups and possibility home delivery of antifungals in consolidation and suppression phases of CM management should be part and parcel of a comprehensive CM/HIV care. These should be incorporated into routine clinical care and included in the local practice guidance, especially in areas with high disease burden.
